# Simultaneous radiosurgery for multiple brain metastases: technical overview of the UCLA experience

**DOI:** 10.1186/s13014-021-01944-w

**Published:** 2021-11-17

**Authors:** Nzhde Agazaryan, Steve Tenn, Chul Lee, Michael Steinberg, John Hegde, Robert Chin, Nader Pouratian, Isaac Yang, Won Kim, Tania Kaprealian

**Affiliations:** 1 Department of Radiation Oncology, David Geffen School of Medicine at UCLA, Los Angeles, CA USA; 2 Department of Neurosurgery, David Geffen School of Medicine at UCLA, Los Angeles, CA USA

## Abstract

**Purpose/objective(s):**

To communicate our institutional experience with single isocenter radiosurgery treatments for multiple brain metastases, including challenges with determining planning target volume (PTV) margins and resulting consequences, image-guidance translational and rotational tolerances, intra-fraction patient motion, and prescription considerations with larger PTV margins.

**Materials/methods:**

Eight patient treatments with 51 targets were planned with various margins using Elements Multiple Brain Mets SRS treatment planning software (Brainlab, Munich, Germany). Forty-eight plans with 0 mm, 1 mm and 2 mm margins were created, including plans with variable margins, where targets more than 6 cm away from the isocenter were planned with larger margins. The dosimetric impact of the margins were analyzed with V5Gy, V8Gy, V10Gy, V12Gy values. Additionally, 12 patient motion data were analyzed to determine both the impact of the repositioning threshold and the distributions of the patient translational and rotational movements.

**Results:**

The V5Gy, V8Gy, V10Gy, V12Gy volumes approximately doubled when margins change from 0 to 1 mm and tripled when change from 0 to 2 mm. With variable margins, the aggregated results are similar to results from plans using the lower of two margins, since only 12.2% of the targets were more than 6 cm away from the isocenter. With 0.5 mm re-positioning threshold, 57.4% of the time the patients are repositioned. Reducing the threshold to 0.25 mm results in 91.7% repositioning rate, due to limitations of the fusion algorithm and actual patient motion. The 90th percentile of translational movements in all directions is 0.7 mm, while the 90th percentile of rotational movements in all directions is 0.6 degrees. Median translations and rotations are 0.2 mm and 0.2 degrees, respectively.

**Conclusions:**

Based on the data presented, we have switched our modus operandi from 2 to 1 mm PTV margins, with an eventual goal of using 0.5 and 1.0 mm variable margins when an automated margin assignment method becomes available. The 0.5 mm and 0.5 degrees repositioning thresholds are clinically appropriate with small residual patient movements.

## Introduction

Radiation therapy treatments for multiple brain metastases patients include whole brain radiotherapy (WBRT), stereotactic radiosurgery (SRS) or hypofractionated radiotherapy for larger targets [1, 2]. Stereotactic radiosurgery for multiple brain metastases has historically been treated with multiple isocenters, typically one isocenter per lesion for linac-based deliveries. Aside from the development of image guided frameless radiosurgery, treatment of multiple brain metastases with a single isocenter is one of the most significant recent technological advances in radiosurgery. A single isocenter treatment technique for multiple brain metastases provides significant improvements in patient experience, increased patient eligibility, reduced planning times, reduced treatment times, increased machine throughput, other economic benefits, and cost effectiveness [3–6]. Bodensohn, et. al reported on the feasibility and safety of single isocenter treatments for patients with multiple brain metastases [7]. In the view of the newly proposed legislation by the Centers for Medicare & Medicaid Services (CMS) on an Alternative Payment Model (APM) affecting Radiation Oncology, the single isocenter treatment technique is a timely and relevant treatment method considering the upcoming bundled reimbursement rates [8].

The purpose of this study is to investigate and communicate some of the challenges associated with the single isocenter radiosurgery treatments and our institutional experience with this technique for multiple brain metastases. The single isocenter treatment technique employs multiple non-coplanar arcs and requires stricter rotational positioning accuracy to minimize the rotational errors that displace targets distant from the isocenter [9–11]. Utilizing larger margins for planning target volumes (PTV) improves target coverage probability; however, larger PTV margins significantly increase the irradiated volume and, thus, normal tissue irradiation [9–11]. This study addresses how planning target volume margins are determined and the consequences of margin size, the translational and rotational tolerances used for image guided patient setup, intra-fraction patient motion during the treatment, and how PTV margins can affect dose prescriptions. Even with the same margins, depending on the prescription and conformity of the plans, institutions may be creating effectively larger margins with more generous distributions or higher prescriptions.

**Fig. 1:**
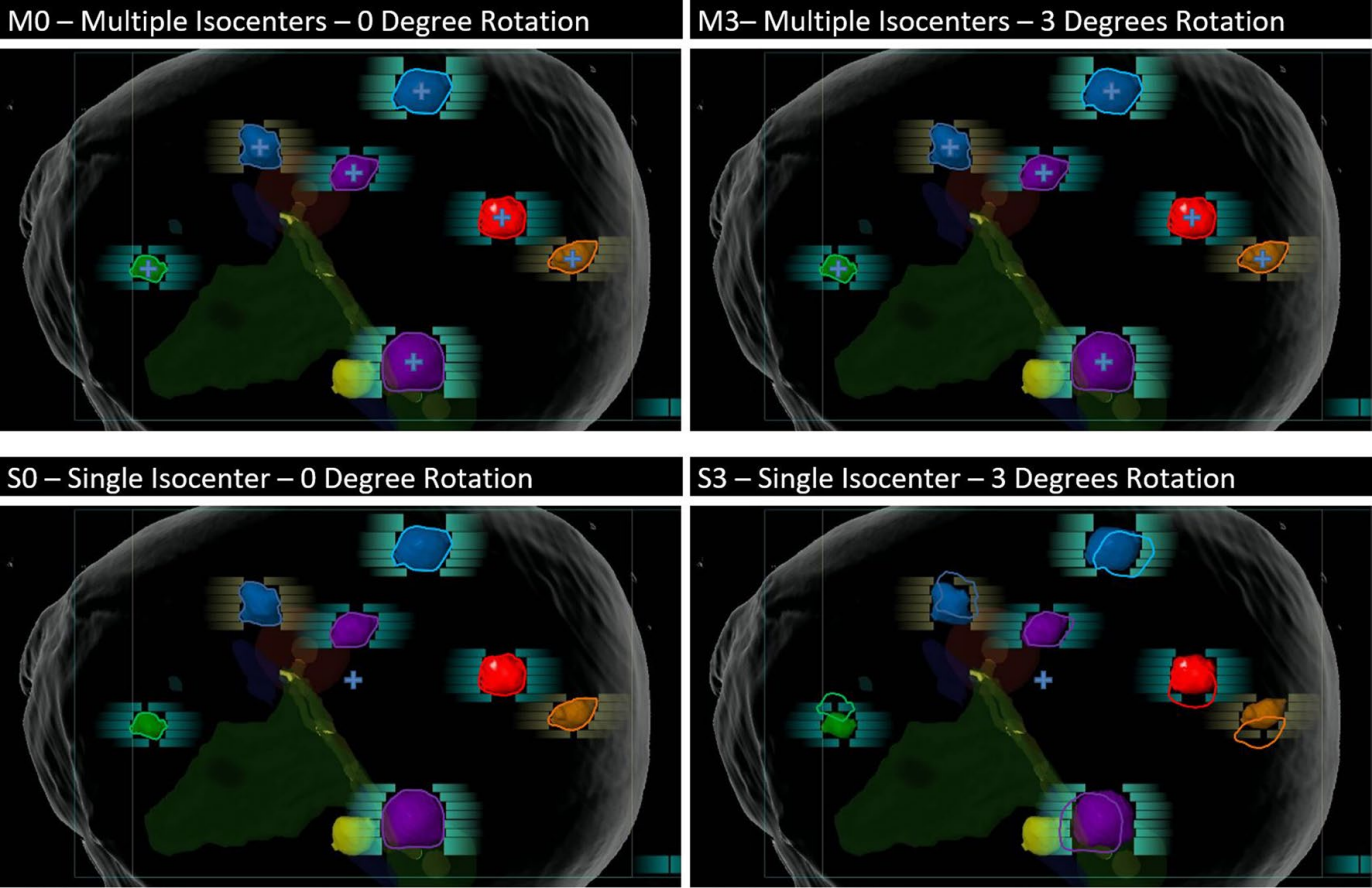
Simulation of 3-degree rotation and the impact of the rotation on each of the targets. The first row is the Multiple Isocenter plan and the second row is the Single Isocenter plan. With a single isocenter treatment plan, the effects of these rotations become much more pronounced

In Fig. 1, we simulate uncorrected 3-degree rotation about the isocenter and the impact of the rotation on each of the targets for a multiple isocenter plan, as well as a single isocenter plan. The first row is the multiple isocenter plan and the second row is the single isocenter plan. With a single isocenter treatment plan, the effects of these rotations become much more pronounced. In fact, some targets can be almost completely missed with single isocenter treatment and a 3-degree misalignment. Appropriate margin selection is critical when treating multiple brain metastases with single isocenter.

Currently, there are two commercially available systems that are task-specific for treating multiple brain metastases with a single isocenter technique: Elements Multiple Brain Mets SRS (Brainlab, Munich, Germany) and HyperArc (Varian Medical Systems, Palo Alto, CA). Elements Multiple Brain Mets SRS treatments are delivered with Enhanced Dynamic Conformal Arcs (DCA), while HyperArc treatments are delivered with Volumetric Modulated Arc Therapy (VMAT) [12]. General-purpose planning systems are also utilized in clinical practice to treat multiple brain metastases with single isocenter. Some examples are Monaco (Elekta, Stockholm, Sweden), Eclipse (Varian Medical Systems, Palo Alto, CA), and RayStation (RaySearch Laboratories, Stockholm, Sweden) [13, 14]. Each of these systems and approaches have their advantages and disadvantages. For example, in one study, a task specific planning tool for single isocenter stereotactic radiosurgery for multiple brain metastases using dynamic conformal arc therapy (DCAT) was compared to a general-purpose volumetric modulated arc therapy (VMAT) [15]. The authors concluded that the task-specific DCAT planning system performed better than the general-purpose VMAT in terms of healthy brain sparing; whereas, VMAT can perform better for irregularly shaped lesions. This study focuses on the clinical use of the Enhanced Dynamic Conformal Arc Therapy system, with the use of the Elements Multiple Brain Mets SRS (MME versions 1.5 and 2.0).

## Materials and methods

This study commenced after the full validation and commissioning of the system. Additionally the dosimetric and geometric quality assurance for our first 12 patients treated in the department consisted of both measurement and independent dose calculation. Measurements were performed with both GafChromic EBT-XD film and pinpoint ion chamber (PTW N31016) in a plastic water phantom (Quick Phantom, Ashland and IMT). Initially, we selected patients with at least one lesion large enough to be accurately measured with an ion chamber. Relative dosimetry with film was then used for all smaller metastases within the same plan. In addition to the measurements, an independent dose re-calculation was performed using a third-party platform (Eclipse, AAA algorithm ver. 13623, Varian Medical Systems, Palo Alto, CA). Our AAA algorithm is commissioned and configured with completely independent data from that used in the MME algorithm.

Patients included in this study were positioned and immobilized with a frameless SRS thermoplastic mask system (Brainlab, Munich, Germany). The system consists of a carbon fiber frame that holds the mask above the treatment couch and two thermoplastic mask halves that are molded around the patient’s head. Patients were scanned on either Siemens Sensation Open or Philips Big Bore RT CT scanner with 1.5 mm and 1 mm slice thickness respectively. Contrast enhanced T1 weighted volumetric MRI scans (MPRAGE) of 1 mm, or better, isotropic resolution were also obtained and fused to the simulation CT scan within MME 1.5 in order to define target volumes. The time intervals between the CT simulation and radiosurgical treatments are less than five days. This is based on the institutional guideline for all patients, published by Agazaryan et.al in their article “The Timeliness Initiative: Continuous Process Improvement for Prompt Initiation of Radiation Therapy Treatment” [16]. The time intervals between the MRI scans and patient treatments are less than 14 days.

Contouring was performed on the MRI images (MPRAGE). Normal tissue structures included brainstem, optic pathway, eyes and lenses, cochlea and the whole brain. The normal tissue structures were auto contoured by the Elements Anatomical Mapping and corrected manually if necessary. Gross tumor volumes (GTV) were contoured by a resident or fellow then reviewed and approved by both a radiation oncologist and an attending neurosurgeon. During the initial phase of our clinical experience, the GTVs were typically expanded 2 mm isotopically to create the PTVs. In some instances where the targets were clustered closely together (not more than 6 cm apart), 1 mm margins were applied to create PTVs.

Plans were created with Multiple Brain Mets SRS Element. This treatment planning software is designed to treat multiple cranial targets with a single isocenter and an associated set of DCA. At our institution, MME is commissioned for use with a Novalis Tx (6 MV and 6 SRS energy modes) and a Truebeam STx (6FFF mode). Both machines utilize the HDMLC (Varian Medical System, Palo Alto, CA) that has 2.5 mm leaf width for the central leaves.

The first cohort of patients consisted of 8 patients. We re-planned these 8 patient treatment plans (51 targets) with various PTV margin sizes using MME 2.0 to study how margin size would affect normal tissue irradiation for patients with multiple brain metastases. Plans with uniform 0 mm, 1 mm and 2 mm margins for all targets were created. In addition, we created plans with tiered margins. Targets from 0 to 6 cm were planned with one margin size and targets further than 6 cm away from the isocenter were planned with a different and larger margin. The dosimetric consequences of these margin combinations in terms of the brain V5Gy, V8Gy, V10Gy, V12Gy volumes were then evaluated based on the results of the 48 treatment plans.

Prescribed dose ranged from 16 to 18 Gy. Anywhere from 2 to 11 arcs were used to create plans. Planning time took between 5 and 30 min depending on the complexity of the case. All plans were reviewed and approved by both a radiation oncologist and a neurosurgeon before being delivered.

Image guidance that corrects for both translation and rotation is essential for safe and accurate treatment with single isocenter multiple target delivery techniques [9]. At our institution, ExacTrac X-ray (Brainlab, Munich, Germany) is used for all cranial radiosurgery treatments. Studies of the ExacTrac X-ray 6DOF (six degrees of freedom) system on phantoms have demonstrated setup accuracy potential better than 0.5 mm for translations and better than 0.2 degrees for rotations [17].

Patients treated with the single isocenter technique were positioned with ExacTrac 6DOF stereoscopic kV imaging (ExacTrac 6.2, Brainlab, Munich, Germany) [18]. CBCT was also obtained following ExacTrac guided setup and prior to beam delivery to verify patient positioning. ExacTrac verification X-rays were then acquired immediately prior to each treatment arc. Deviations larger than 0.5 mm or 0.5 degrees were corrected before a beam could be delivered. The treatments were managed with ARIA Record and Verify (RV) System (ARIA 13.6, Varian Medical Systems, Palo Alto, CA).

The second cohort of the patients consisted of 12 patients. Patient movement during 12 single isocenter SRS treatment deliveries was obtained from the ExacTrac system. ExacTrac stores the translation and rotation displacements (relative to the isocenter) that are detected each time X-ray correction or verification is performed. These displacements, along with the known locations of each tumor relative to the isocenter, were used to determine the total displacement magnitude for each tumor. We reviewed the distribution of patient translations and rotations and the associated target displacement statistics as a function of distance from the isocenter. These distributions determine the PTV margins required for planning with our current setup tolerances (0.5 mm and 0.5 degrees). We also studied the frequency of patient repositioning for our current setup tolerances, as well as the predicted frequency if a tighter translational tolerance of 0.25 mm were applied.

**Fig. 2:**
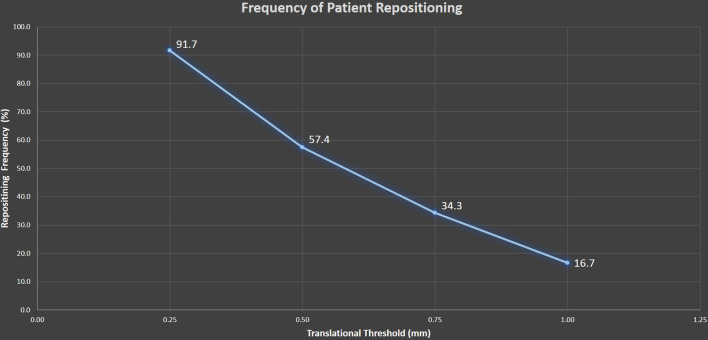
ExacTrac displacement data from a cohort of 12 patients were analyzed. With the 0.5 mm threshold, 57.4% of the time the patients are repositioned after the ExacTrac images

**Fig. 3:**
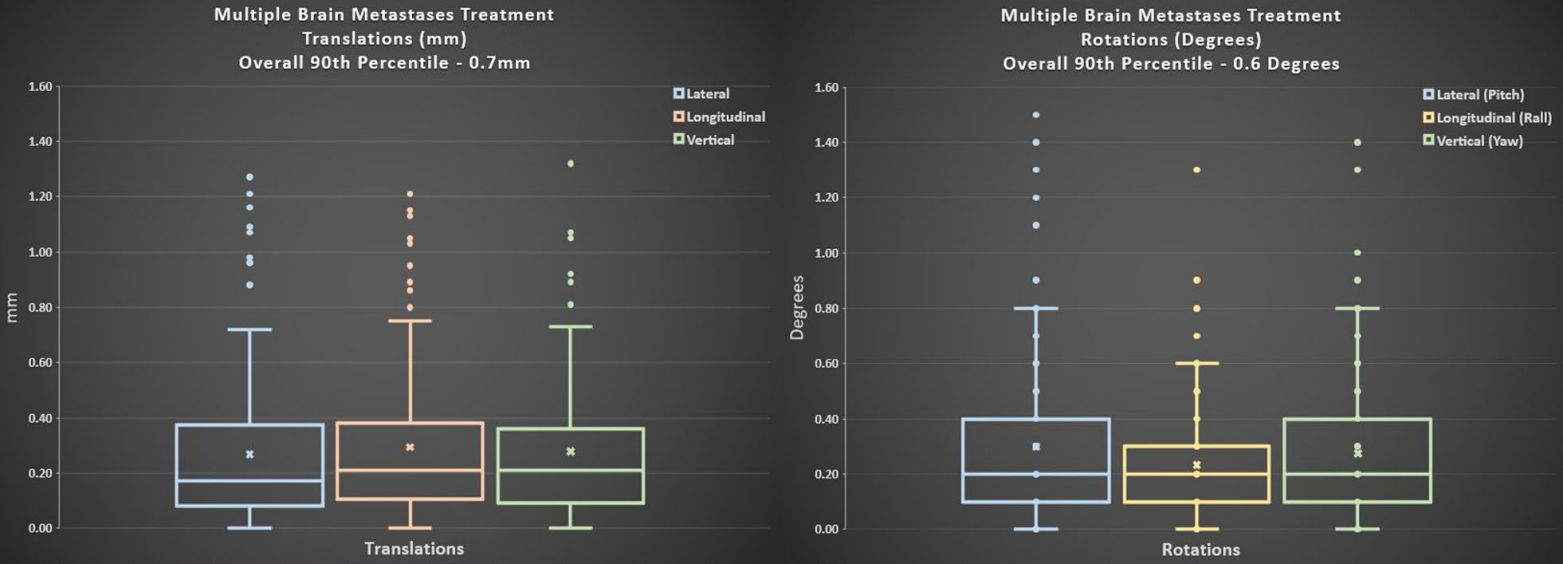
Patient data from a cohort of 12 patients were analyzed for the translational movements. 90th percentile of translational movements in all directions is 0.7 mm with median translations of 0.2 mm. 90th percentile of translational movements in all directions is 0.6 degrees with median rotations of 0.2 degrees

## Results

**Fig. 4:**
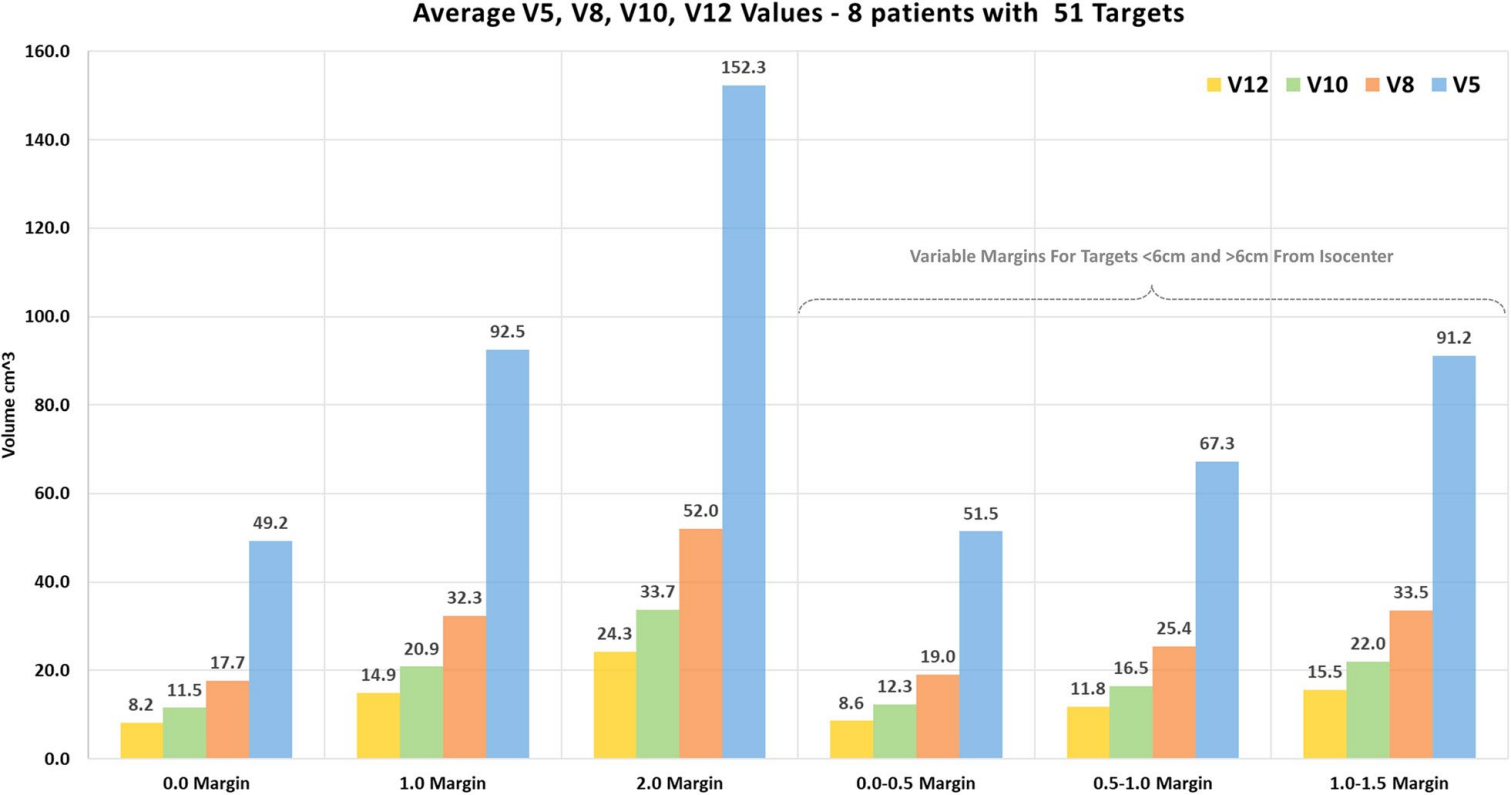
The dosimetric impact of the margins are shown for a cohort of 8 patients with 51 targets. Overall, 48 plans were created using various margins and V5Gy, V8Gy, V10Gy, V12Gy values were analyzed

The data from 8 patient treatments with 51 targets were included in the dosimetric study. A total of 48 plans with 0 mm, 1 mm, 2 mm and variable margins were created and summarized in the Fig. 4. The V5Gy, V8Gy, V10Gy, V12Gy volumes approximately doubled when margins change from 0 to 1 mm and tripled when change from 0 to 2 mm. With variable margins, while the irradiated volumes get smaller in terms of the V5Gy, V8Gy, V10Gy, V12Gy, the aggregated results are similar to results from plans using the lower of two margins, since only 12.2% of the targets were more than 6 cm away from the isocenter.

The analysis of ExacTrac data from the second cohort of 12 patients shows that with the 0.5 mm threshold, 57.4% of the verification images required patient repositioning (Fig. 2). Additionally, the data shows that reducing the threshold to 0.25 mm will result in a 91.7% repositioning rate, where some of the increase in the repositioning rate is due to limitations of the fusion algorithm and some of it due to actual patient motion.

The same patient data was analyzed for the distributions of the translational and rotational residual movements. The 90th percentile of translational movements in all directions is 0.7 mm, while the 90th percentile of rotational movements in all directions is 0.6 degrees. Median translations and rotations are 0.2 mm and 0.2 degrees (Fig. 3).

**Fig. 5:**
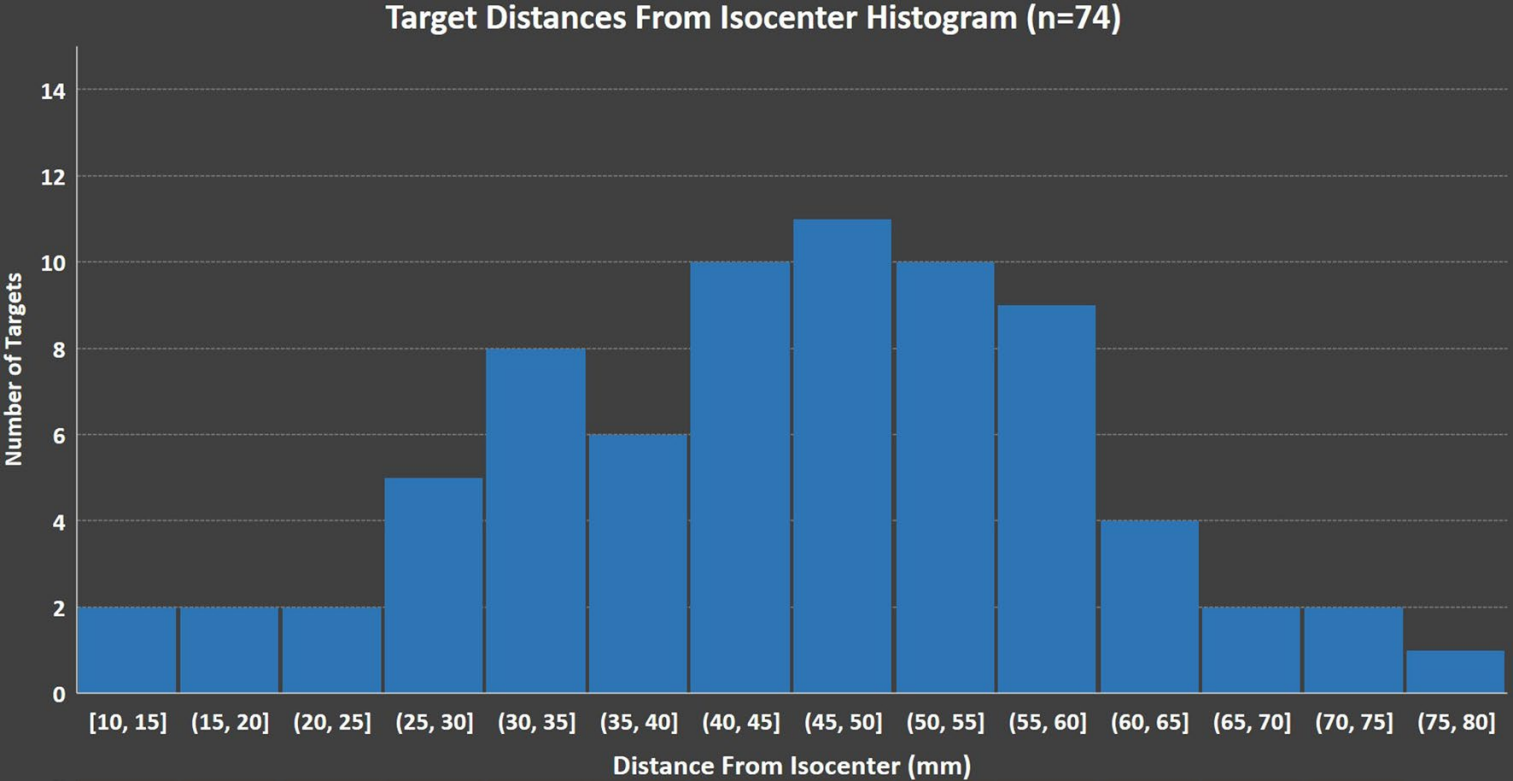
Histogram of targets binned by the distance from the isocenter. The data shown may further support the use of the variable margins, since a small fraction of targets are more than 6 cm away from the isocenter

For this cohort of 12 patients, we have compiled and analyzed the distances of targets from the treatment isocenter and found that 12.2% for the targets are more than 6 cm away. In Fig. 5, we show a histogram of targets binned by the distance from the isocenter.

## Discussion

As discussed in the previous sections, multiple methods have been employed to validate the dosimetric and geometric accuracy of the system prior to clinical use of the system. MME was validated to be locationally and dosimetrically accurate for clinical use. For the first 12 cases treated, we have performed extensive phantom measurements, including a validation using a 3D printed RTsafe phantom (RTsafe PC, Athens, Greece). Using patient’s skull and internal anatomical bony structures, RTsafe prints a phantom that is filled with polymer gel dosimeter material. This method allows for 3D dosimetric validation. Given that the planning and delivery methods are dynamic conformal arcs, our institutional policies now allow for reduction of the patient specific quality assurance to secondary independent calculations.

Our institution initially treated multiple metastasis with single isocenter using 2.0 mm PTV margins. However, based on the analysis presented, we now apply a 1.0 mm PTV margin. Our goal is to use 0.5 mm margins for targets that are 6 cm or closer to the isocenter and 1.0 mm for targets that are further away. In Fig. 4, the dosimetric impact of the margins are shown for 8 patients with 51 targets. Overall, 48 plans were created using various margins and V5Gy, V8Gy, V10Gy, V12Gy values were analyzed.

The use of variable margins is an important consideration, because in clinical practice, only small portion of targets are far away from the isocenter. The 6 cm criteria is suggested based on the fact that 0.5 degrees rotational misalignment for that distance results to 0.52 mm misalignment; however, other criteria can be used until there is clinical evidence on the use of margins for these types of treatments. Using uniform large margins are suboptimal, in terms of normal tissue sparing, when only a small fraction of the targets in our practice are further away than 6 cm. The data shown in Fig. 5 also supports the use of the variable margins.

**Fig. 6:**
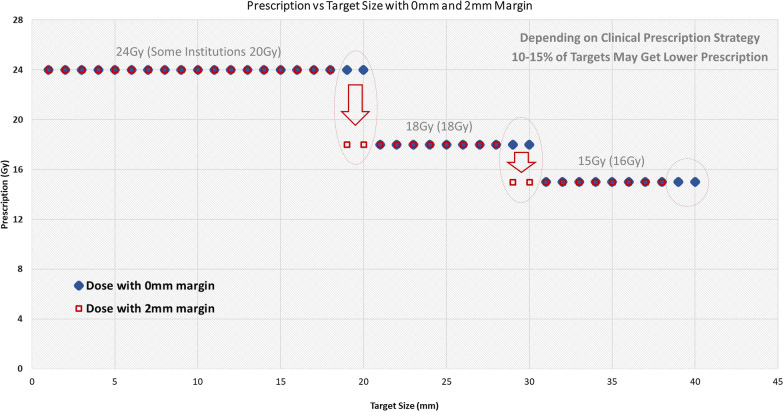
Depending on the clinical prescription strategy, if the target size is strictly followed for determining the prescription levels, then larger margins may also have an impact on prescriptions

Using unnecessarily large margins can also impact the prescription dose if one follows the RTOG 9508 trial guidelines [19]. Depending on the clinical prescription strategy, if the target size is strictly followed for determining the prescription levels, then margin size may also have an impact on prescriptions. For those 10–15 percent of the targets that are at the higher level of the prescription thresholds, these may get lower prescriptions because of the use of larger margins (Fig. 6).

The single isocenter treatment technique includes multiple non-coplanar arcs and requires stricter rotational positioning accuracy to minimize the rotational errors that displace targets distant from the isocenter. Utilizing larger margins for PTVs ensures target coverages; however, larger PTV margins significantly increase the target coverage volume; as well as the normal tissue irradiation. Even with the same margins, depending on the prescription and conformity of the plans, institutions may be creating effectively larger margins with more generous distributions or higher prescriptions.

Our institution initially treated multiple metastasis with single isocenter using 2 mm margins. However, based on the analysis presented, we have now switched to a 1 mm margin. Our eventual goal is to use 0.5 mm margins for targets that are 6 cm or closer to the isocenter and 1.0 mm for targets that are further away when an automated method of margin assignments becomes available.

Treatment delivery times from initial setup until the final arc has been delivered have been determined for these patients using the treatment record as well as recording the typical amount of time needed to acquire the initial ExacTrac images and adjust a patient's position. Our previously published data collected for 22 patients who underwent single isocenter treatments showed a mean treatment time of 30.2 min compared to multiple isocenter treatments with mean treatment time of 75.2 min. We have also published that treatment times for the multiple isocenter technique increased substantially with the number of lesions (11.8 min/lesion), but to a much lesser degree for the single isocenter technique (1.78 min/lesion) [20].

Limitation of this study may be the number of patients analyzed for the margin analysis and patient motion data. It is important to note that the concept and results presented here are unlikely change to any significant degree by adding more patient data. The V5Gy, V8Gy, V10Gy, V12Gy volumes approximately double when margins change from 0 to 1 mm and triple when 2 mm margins are used. Although not presented here, we have briefly modeled the concept in a mathematical way and the results are very similar.

## Conclusions

The Single Isocenter treatment technique enables faster and efficient treatment planning and faster and efficient treatments. Successful implementation of this technique requires accurate positioning and intra-fraction motion management. ExacTrac data from this study suggests that the use of the 0.5 mm and 0.5 degrees thresholds for stereoscopic imaging prior to each arc are clinically appropriate with relatively small residual movements. This result is consistent with our prior larger scale observations for trigeminal neuralgia patients.

Based on the data presented, we have switched our modus operandi from 2 to 1 mm PTV margins, with an eventual goal of using 0.5 and 1.0 mm variable margins when an automated margin assignment method becomes available.

## Data Availability

The datasets generated during and/or analyzed during the current study are not publicly available in adherence to institutional policies on data sharing. The data sharing requests will have to be vetted with the institution.
